# Multisensory stimuli shift perceptual priors to facilitate rapid behavior

**DOI:** 10.1038/s41598-021-02566-8

**Published:** 2021-11-29

**Authors:** John Plass, David Brang

**Affiliations:** Department of Psychology, University of Michigan, Ann Arbor, MI 48109 USA

**Keywords:** Sensory processing, Human behaviour

## Abstract

Multisensory stimuli speed behavioral responses, but the mechanisms subserving these effects remain disputed. Historically, the observation that multisensory reaction times (RTs) outpace models assuming independent sensory channels has been taken as evidence for multisensory integration (the “redundant target effect”; RTE). However, this interpretation has been challenged by alternative explanations based on stimulus sequence effects, RT variability, and/or negative correlations in unisensory processing. To clarify the mechanisms subserving the RTE, we collected RTs from 78 undergraduates in a multisensory simple RT task. Based on previous neurophysiological findings, we hypothesized that the RTE was unlikely to reflect these alternative mechanisms, and more likely reflected pre-potentiation of sensory responses through crossmodal phase-resetting. Contrary to accounts based on stimulus sequence effects, we found that preceding stimuli explained only 3–9% of the variance in apparent RTEs. Comparing three plausible evidence accumulator models, we found that multisensory RT distributions were best explained by increased sensory evidence at stimulus onset. Because crossmodal phase-resetting increases cortical excitability before sensory input arrives, these results are consistent with a mechanism based on pre-potentiation through phase-resetting. Mathematically, this model entails increasing the prior log-odds of stimulus presence, providing a potential link between neurophysiological, behavioral, and computational accounts of multisensory interactions.

## Introduction

**Figure 1:**
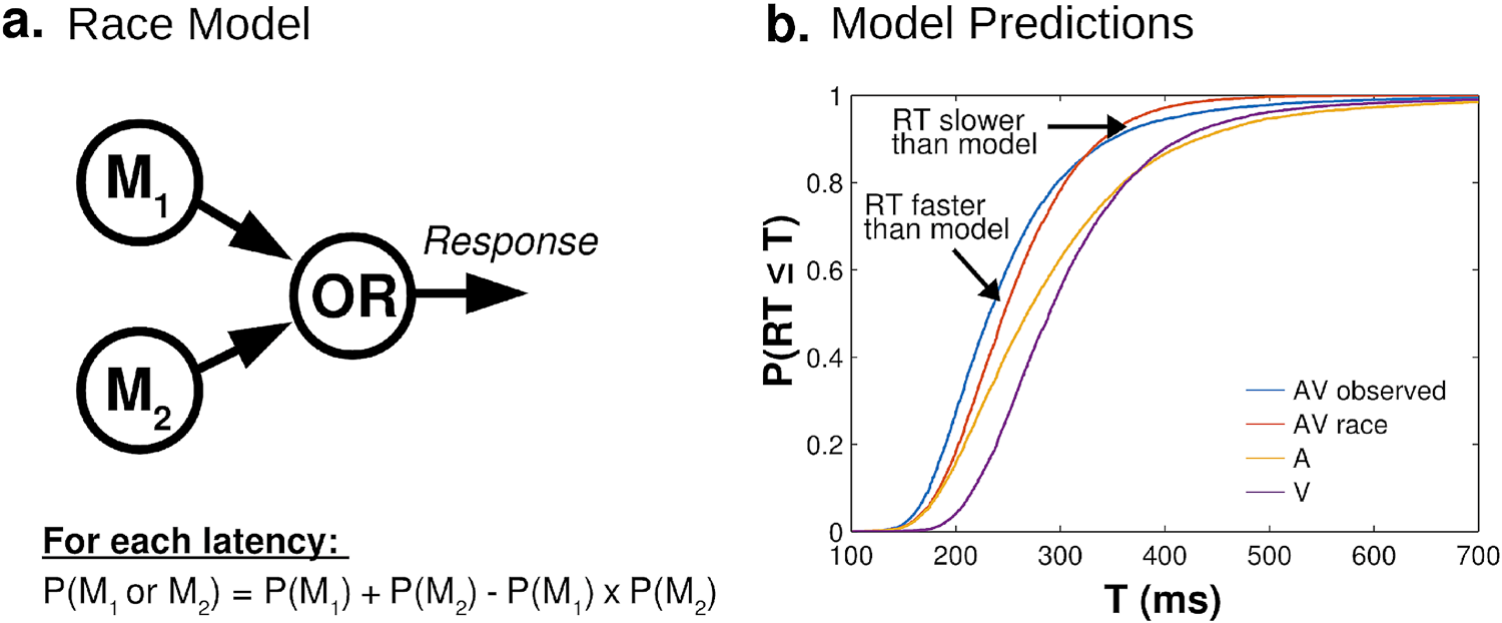
The race model of multisensory reaction times. (**a**) Schematic diagram of the race model architecture. Independent channels for each sensory modality (M_1_ and M_2_) feed into an OR operator which triggers responses. This results in the faster of the two channels triggering a response on any given trial. Assuming no correlation between channels, the race model predicts the probability of a response at some time *T* or sooner using the statistical definition of the OR operator (see equation). (**b**) Race model estimates (red) and empirical RT distribution (blue) for audiovisual (“AV”) trials in the current dataset. The race model estimate for each time bin is estimated using the cumulative reaction time distributions for unisensory auditory (“A”, yellow) and visual (“V”, purple) trials. The race model underestimates the probability of faster trials, indicating that the fastest RTs (left tail) outpace the race the model. However, the race model also overestimates the probability of slower trials, indicating that the slowest RTs (right tail) are slower than race model predictions.

Multisensory stimulation can speed behavioral responses, but the mechanisms responsible for these enhancements remain disputed. A common benchmark used to test for multisensory interactions in behavior is the race model, which gives the expected distribution of simple reaction times when unisensory channels are processed independently, with responses driven by the faster channel on each trial (see Fig. 1)^1–4^. Over the last 35 years, the repeated observation that multisensory reaction times often outpace race model predictions has been taken as evidence for facilitative multisensory interactions in perceptual processing (the “redundant target effect,” RTE)^3,4^. Most commonly, race model violations have been interpreted as evidence for convergence or coactivation in multisensory processing streams, potentially driven by additive or superadditive neural integration of unisensory signals (e.g.,^2,5^; see^3,6^ for review). However, more recently, this facilitative account has been challenged by multiple conflicting observations.

### Alternative explanations for the RTE

#### Sequence effects

First, multiple authors have shown that typical estimates of the RTE are at least partially confounded by sequence effects produced by “modality switch costs”^7–12^. These interference effects occur when a target stimulus is preceded by a stimulus of a different modality on the previous trial (e.g., a visual stimulus preceded by an auditory stimulus), leading to slower RTs than when the stimulus is preceded by a stimulus of the same modality^13^. Indeed, studies have demonstrated reduced race model violations when modality switches are removed from an auditory–visual paradigm^7–10^.

These sequence effects may exaggerate apparent RTE effects because modality switch costs are more likely to slow RTs on unisensory trials (which are used to estimate the race model) than on multisensory trials (which are compared to the race model). Therefore, observed race model violations may at least partially reflect increased switch costs in the unisensory conditions, rather than multisensory facilitation in the multisensory condition.

For example, in a typical RTE experiment with randomly-ordered auditory (“A”), visual (“V”), and audiovisual (“AV”) trials, AV trials will always include at least one target signal that was present in the previous trial. However, unimodal A or V trials will not include any repeated signals on ~ 1/3 of trials (i.e., when A precedes V, or V precedes A), leading to increased modality switch costs on unisensory trials. Because these switch costs are not typically accounted for in race model estimates, they may produce apparent differences between multisensory RTs and race model estimates that are not actually driven by multisensory facilitation.

**Figure 2:**
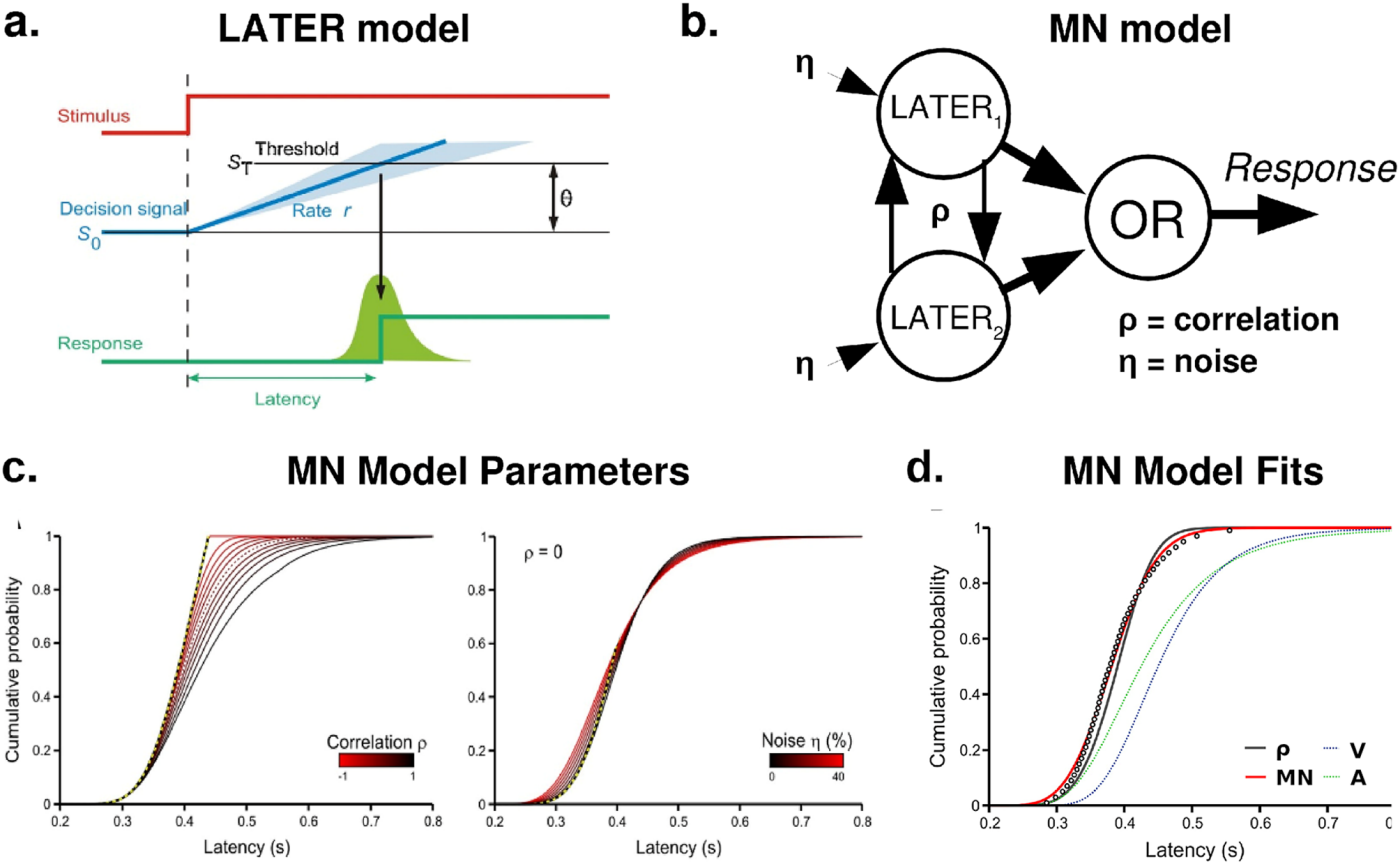
The LATER model^14^ and its multisensory extension. (**a**) Schematic diagram of the (unisensory) LATER model. Evidence accumulation begins at S_0_ and accumulates at rate *r* towards the response threshold S_T_. The rate parameter, *r*, is modelled as a normal distribution, resulting in a reciprocal normal (recinormal) reaction time distribution (green distribution), Θ/*N*(μ,σ^2^), where the distance-to-threshold parameter, Θ, is set to 1 by convention. (**b**) Schematic diagram of the multisensory noise (MN) model^9^. Two unisensory LATER models with correlation ρ and added noise η feed into an OR operator which triggers responses. (**c**) Effects of the ρ (left) and η (right) parameters on predicted cumulative RT distributions. Correlations primarily affect RTs towards the right end of the distribution, while added noise primarily influences RTs towards the left end of the distribution. (**d**) The MN model (red) with negative correlations and increased noise provides an excellent fit for empirical audiovisual RT distributions, surpassing the fit produced by accounting for intersensory correlations alone (ρ, black). Panel A modified with permission from^14^. Panels C and D modified with permission from^9^.

Specifically, switch costs may introduce negative dependencies in processing times across sensory modalities, but these dependencies are not accounted for in standard race model estimates^9^. Because negative dependencies increase statistical facilitation in race models (see Fig. 2c, left), race model formulations assuming statistical independence between sensory modalities may underestimate race model predictions, potentially producing false-positive race model violations.

For example, when an AV trial follows an A stimulus (“A–AV”), the repeated A signal may be expected to be processed faster than the non-repeated V signal due to modality switch costs. Similarly, on V–AV trials, the V signal may tend to be processed faster than the A signal. Thus, on bimodal trials in which one stimulus is repeated and the other is not, unimodal processing times may become negatively correlated due to the effects of modality switch costs. If this is the case, an appropriate model of trial-by-trial “races” between unimodal RT distributions should account for these negative dependencies: on bimodal trials affected by switch costs, RTs for the repeated stimulus should be sampled from the faster end of the distribution, while RTs for the non-repeated stimulus should be sampled from the slower end. However, race model estimates assuming statistical independence between sensory modalities do not account for these negative dependencies, effectively sampling at random from each unimodal RT distribution regardless of whether each modality was repeated or not. Thus, to the extent that modality switch costs affect processing times on bimodal trials, race models assuming statistical independence may produce misleadingly slow RT predictions.

#### Effects of correlation and noise

Second, the observation that multisensory RTs are both faster and slower than race model predictions in different parts of the RT distribution has been taken as evidence against a facilitative account of race model violations^9^. Although the fastest multisensory RTs (left tail of RT distribution) tend to be faster than race model predictions, the slowest multisensory RTs (right tail of RT distribution) tend to be slower than predicted (see Fig. 1b). This observation has led to the alternative hypothesis that multisensory stimulation does not result in RT enhancements but, rather, an increase in RT variability^9^. Because an increase in variability flattens the “slope” of the RT cumulative distribution function (CDF), it can account for both positive and negative deviations from race model predictions.

### The multisensory noise (MN) model

Using neurally inspired linear evidence accumulator models (LATER model, Fig. 2a)^14^, Otto and Mammasian showed that these deviations from race model predictions can be accounted for by a combination of negative correlations (Fig. 2c, left) and increased variability (Fig. 2c, right) in evidence accumulation rates during multisensory stimulation. We refer to this model as the multisensory noise (MN) model (Fig. 2b)^9^. In this model, negative correlations between unisensory evidence accumulation rates account for stimulus sequence effects (i.e., switch costs), while increased variability accounts for further deviations from the correlation-corrected race model. Fitting the model with two free parameters for (intersensory correlation, ρ, and added noise, η) produced very good fits for multisensory RT CDFs (Fig. 2d), suggesting that race model violations may reflect the combined effects of negative intersensory correlations and increased variability in evidence accumulation rates. However, the MN model may be inconsistent with neurophysiological findings on crossmodal interactions subserving the RTE.

### The crossmodal pre-potentiation (CP) model

A growing body of research suggests that multisensory stimulation enhances neural responses through crossmodal phase-resetting of intrinsic oscillations in sensory cortex^15–22^, and that RT speeding in the RTE is associated with the strength of crossmodal and sensorimotor phase-alignment produced by multisensory stimulation^23,24^. These effects are referred to as “crossmodal” because they involve stimuli from one sensory modality modulating activity in brain regions or individual neurons that are primarily responsive to another sensory modality^20^.

Contrary to the MN model's predictions, such phase-alignment is expected to result in *decreased* variability and *increased* crossmodal correlations in multisensory processing times. By shifting membrane potentials closer to spiking thresholds, phase-resetting places sensory neurons in a transient high-excitability state that increases neural sensitivity and speeds neural responses to temporally aligned input^20,25,26^. In the context of multisensory stimulation, this also results in decreased variability in unisensory processing times, as evidenced by increased inter-trial phase coherence (ITPC) in nominally “unisensory” cortex during multisensory stimulation^15–17,19,21^. This increase in ITPC is also associated with increased phase-locking between oscillations in sensory and motor cortex, suggesting that these multisensory enhancements are propagated to motor regions^24^. Thus, the neurophysiological evidence suggests that multisensory stimulation would be expected to decrease, rather than increase, variability in sensorimotor processing times.

Crossmodal phase-resetting also produces temporal dependencies between unisensory processes by aligning periods of heightened excitability across unisensory cortices^20,21,25^. These intersensory dependencies would be expected to produce positive correlations in evidence accumulation rates by aligning cortical states across sensory cortices. Thus, contrary to the MN model's predictions, the extant neurophysiological literature predicts that the RTE would be associated with *decreased* response variability and *positive* intersensory correlations.

Here, we hypothesized that the expected effects of crossmodal phase-resetting could provide an alternative explanation for the unique shape of bimodal RT CDFs. Because crossmodal phase-resetting transiently shifts membrane potentials closer to spiking thresholds, it pre-potentiates spiking responses in low-level sensory cortex when sensory input arrives^20,21,25^. Under the plausible assumption that sensory evidence is encoded in spike rates^14^, this pre-potentiation can be thought of as decreasing the distance between the initial evidentiary state, S_0_, and the response threshold, S_T,_ (i.e., decreasing the distance-to-threshold parameter, Θ) in the LATER model (Fig. 2a). That is, increasing S_0_ (or, equivalently, decreasing S_T_) is analogous to pre-potentiating neural evidence accumulation by pre-emptively shifting membrane potentials closer to their spiking threshold.

**Figure 3:**
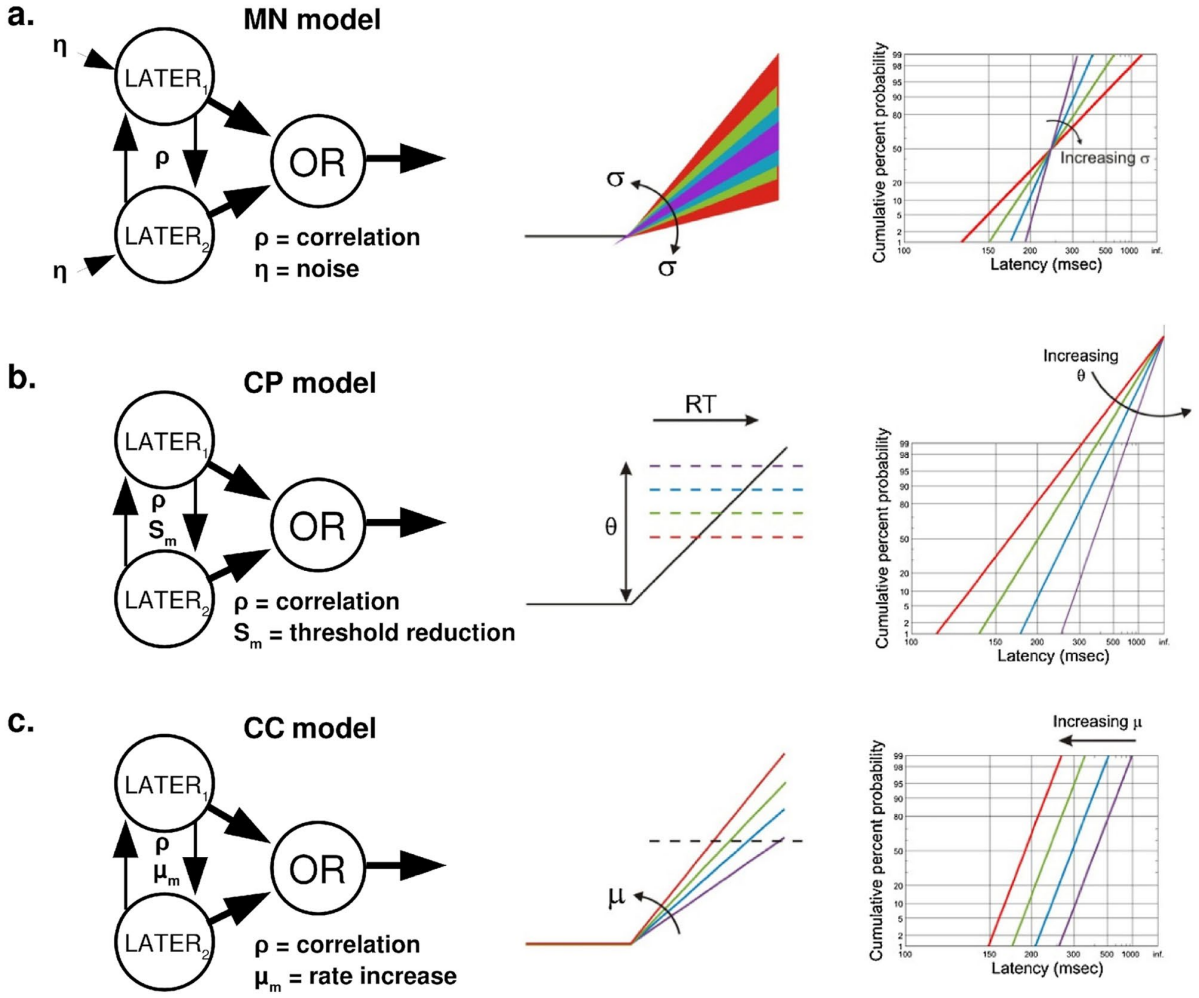
Schematic diagrams of the models compared in this article. Leftmost panels show how unisensory LATER models interact in each multisensory model. Center panels show how unisensory evidence accumulation is affected by each type of multisensory interaction. Rightmost panels show how RT distributions are affected by these changes in evidence accumulation. RT distributions are plotted on reciprobit axes (abscissa: reciprocal; ordinate: probit)^14^. (**a**) In the multisensory noise (MN) model, increased noise (σ + η) increases variability in the rate of evidence accumulation (center), causing the RT distribution to “swivel” about the average accumulation rate (right). (**b**) In the crossmodal pre-potentiation model (CP), decreasing the distance-to-threshold parameter (Θ − S_m_) shifts the response threshold downward (center), causing the RT distribution to “swivel” about the rightmost extreme. (**c**) In the crossmodal co-activation model (CC), increasing the mean rate of evidence accumulation (μ + μ_m_) increases the rate at which the distance-to-threshold Θ is traversed (center), causing the RT distribution to translate leftward. Center and right panels modified with permission from^14^.

As shown in Fig. 3, when plotted on reciprobit axes (abscissa: reciprocal; ordinate: probit), both the σ parameter (Fig. 3a, right) and the Θ parameter (Fig. 3b, right) cause the RT CDF to “swivel,” but about a different point^14^. The σ parameter causes swiveling about the median, while the Θ parameter causes swiveling about the rightmost extreme. Thus, shifts in either parameter could explain the observed flattening of the “slope” of bimodal RT CDFs relative to race model predictions. However, while increasing the σ parameter can explain the additional slowing of slower RTs relative to the race model, decreasing the Θ would not.

We hypothesized that this RT slowing could be explained by intermodal correlations produced by phase coherence across sensory cortices. As shown in Fig. 2b, positive intersensory correlations reduce RTs primarily towards the right end of the distribution. Thus, we predicted that the combined effects of reducing Θ (through crossmodal pre-potentiation) and increasing the correlation coefficient, ρ, (through crossmodal phase coherence) could explain the observed shape of bimodal RT CDFs. We refer to this model as the crossmodal pre-potentiation (CP) model. Further details regarding the parameters of the LATER model can be found in Noorani and Carpenter^14^.

### The crossmodal co-activation (CC) model

In addition, we considered the possibility that multisensory interactions could increase the mean rate of evidence accumulation, μ. In contrast to the CP model predictions, this would entail faster evidence accumulation over time, rather than an initial increase in sensory evidence at onset. This results in leftward shift of the RT CDF (Fig. 3c, right).

Such an effect would be physiologically consistent with crossmodal co-activation (CC), in which multisensory stimulation produces increased spiking rates relative to unimodal stimulation. Neurally, such an effect could be mediated by additive or superadditive spiking responses to multisensory stimuli, such as those observed in the superior colliculus (SC)^27,28^. While, to our knowledge, there is no direct neural evidence of the SC’s involvement in the RTE, some authors have hypothesized that the SC may subserve the RTE due to its known multisensory function (e.g.,^29^). This hypothesis is potentially supported by behavioral studies showing that the RTE is strengthened for stimuli which the SC is known to be sensitive to^29–31^. However, this interpretation may be challenged by the observation that the RTE occurs even when multisensory stimuli are spatially separated far beyond the expected binding window of the SC^32^.

### The current study

Here, we sought to clarify the mechanisms underlying the RTE by explicitly estimating the proportion of RTE variance explained by modality switch costs and by comparing the three aforementioned models of multisensory RT distributions. Towards this end, we collected reaction time data from a large sample of participants (N = 78) who completed a simple reaction task including auditory (A), visual (V), tactile (T), and bimodal (AV, AT, VT) target stimuli (median RT and accuracy for each condition shown in Supplemental Fig. S1).

Based on the neurophysiological evidence reviewed above, we hypothesized that the RTE was unlikely to be explained entirely by sequence effects (i.e., modality switch costs) or increased noise, and more likely reflected crossmodal pre-potentiation through phase-resetting.

Our results suggest that, indeed, stimulus sequence effects only weakly influence the RTE, and do not change the overall shape of bimodal RT CDFs. Moreover, our model comparisons suggest that the RTE is best modelled by a combination of response threshold reduction and positive intersensory correlation. This result is consistent with a mechanism based on crossmodal pre-potentiation through phase-resetting, and mathematically represents shifting the system's prior log odds to favor stimulus presence versus absence. Thus, this model may provide an important link between neurophysiological, behavioral, and computational accounts of multisensory interactions.

## Results

### The redundant target effect (RTE)

**Figure 4:**
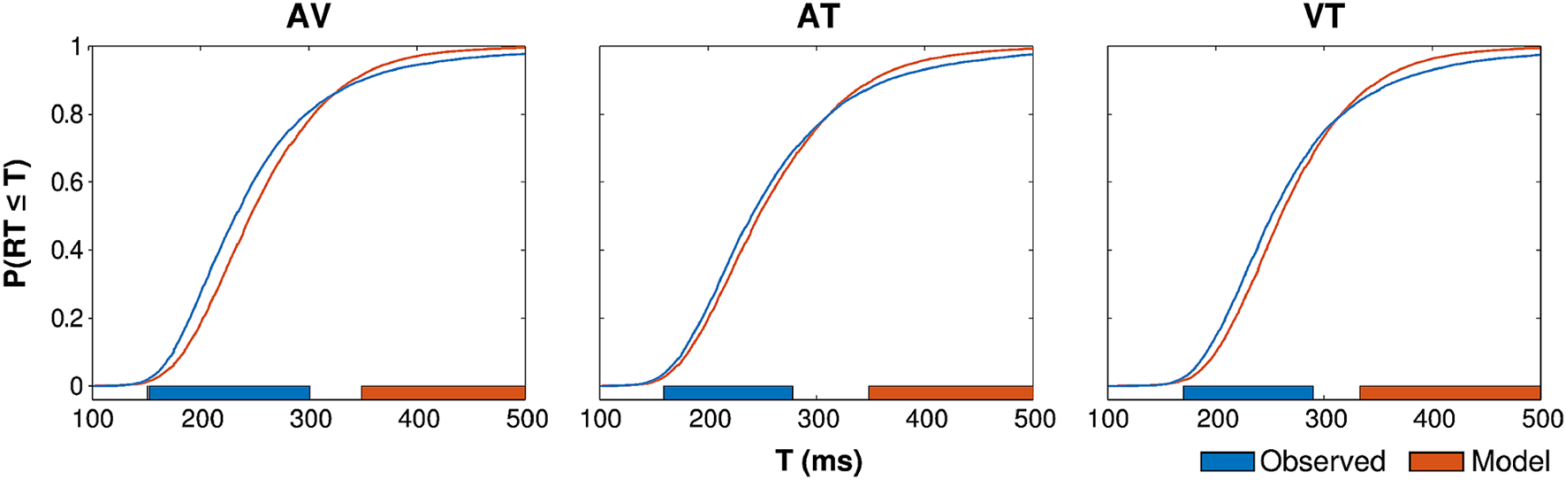
Comparisons of race model predictions (orange) and empirical RT distributions (blue) for audiovisual (“AV”), audiotactile (“AT”), and visuotactile (“VT”) trials. Colored bars at the bottom of each graph indicate time bins where the data and model differed significantly (*p* < .05) after FWE correction. Blue bars indicate higher cumulative probability in the observed RT distribution, and orange bars indicate higher cumulative probability in the race model.

First, to verify that the typical RTE was observed in our data, we computed race models for the AV, AT, and VT conditions and compared them to participants' empirical RT CDFs. Race models and RT CDFs were computed individually for each subject and then averaged for display (Fig. 4). Averaged unimodal RT CDFs are shown with corresponding bimodal RT CDFs in Supplemental Fig. S2.

To test for significant differences between model predictions and empirical CDFs, we used a non-parametric permutation test (sign-flipping). For each 1 ms time bin in each condition, we performed a paired *t*-test, and compared the observed *t* statistics for each bin to the distribution of maximum *t*-statistics (across all bins) for 10,000 permutations. Importantly, for each permutation, the same sign was applied to all time bins across all three conditions (AV, AT, and VT) for each subject, so that dependencies across bins and conditions were preserved in the estimated null distribution. This procedure controls the family-wise error (FWE) rate across all tests while taking into consideration potential dependencies across time bins and conditions^33,34^. We used the absolute value of each *t* statistic to perform two-tailed tests.

Figure 4 shows the average race model prediction (orange) and average empirical CDF (blue) for each condition. Colored bars at the bottom of each graph indicate time bins where the data and model differed significantly (*p* < .05) after FWE correction, with blue bars indicating higher cumulative probability in the observed RT distribution, and orange bars indicating higher cumulative probability in the race model. Consistent with the observations of Otto and Mamassian^9^, the fastest multisensory RTs (left tail of RT distribution) were significantly faster than race model predictions in all three conditions, while the slowest multisensory RTs (right tail of RT distribution) were significantly slower than predicted.

### Effects of previous stimuli

To examine potential effects of preceding stimuli on observed race model violations, we computed separate CDFs for each two-stimulus sequence, using only trials with the same preceding stimulus to compute each race model and bimodal RT CDF. This approach effectively controls for lag-1 sequence effects by ensuring that potential effects of the previous stimulus are incorporated into both the race model and empirical bimodal RTs. Importantly, this ensures that, for conditions potentially influenced by modality switch costs, race models are estimated using only unimodal trials that are influenced by those same switch costs. For example, for AV trials preceded by A trials (“A–AV”), race models are computed using only A–A (repeated modality) and A–V (non-repeated modality) trials. Similarly, for V–AV trials, race models are computed using only V–A (non-repeated modality) and V–V (repeated modality) trials. This procedure effectively removes the potential confounding effects of modality switch costs by ensuring that race models are only estimated using unimodal RT distributions that would be appropriately paired under the assumption of switch cost effects (i.e., in this example, one repeated and one non-repeated modality), and not those which would not be paired under these assumptions (i.e., in this example, two repeated or two non-repeated modalities). Statistical inference was performed using the same permutation testing procedure as above, correcting for multiple comparisons across all time bins and stimulus sequences.

**Figure 5:**
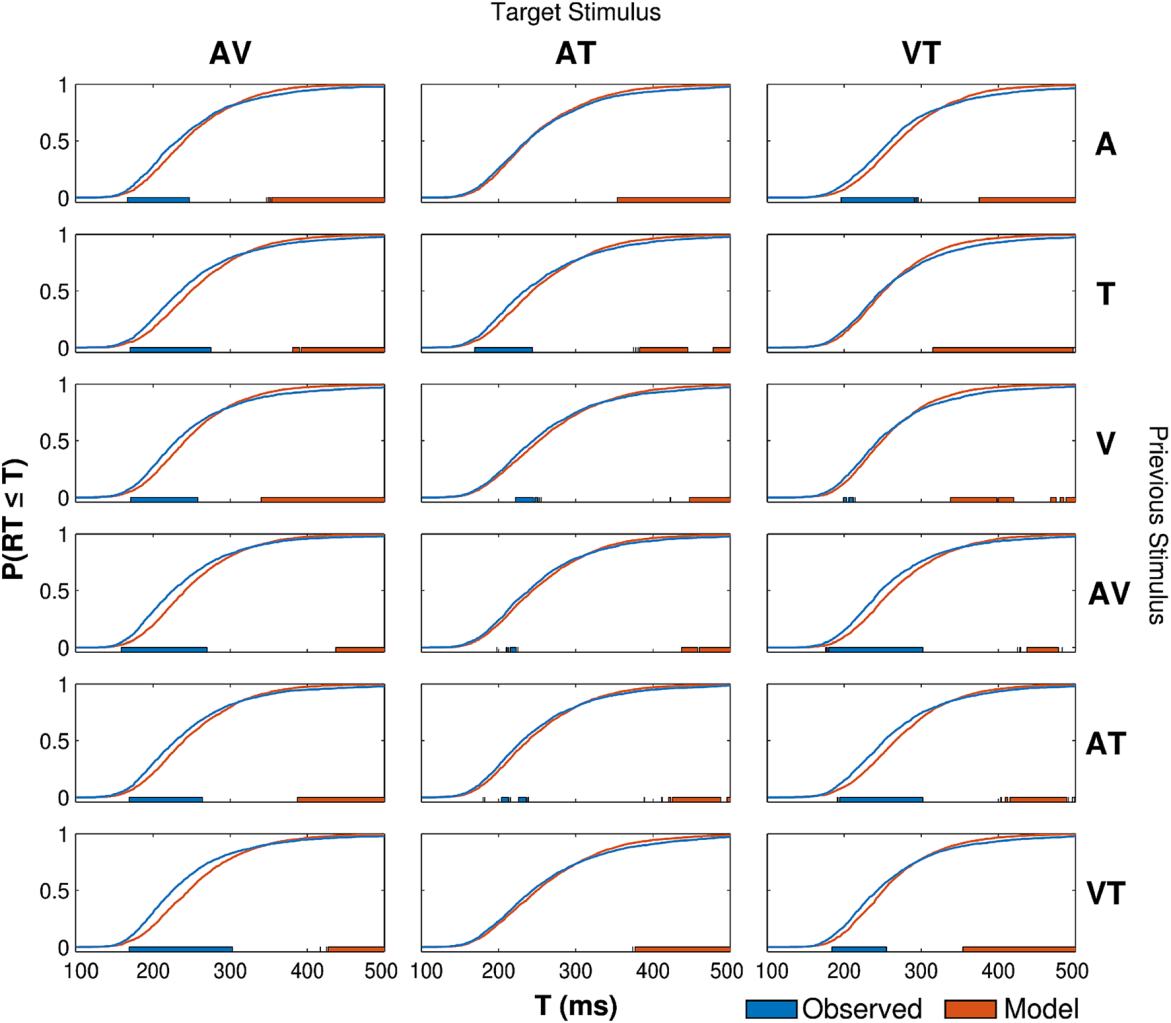
Comparisons of race model predictions (orange) and empirical RT distributions (blue) for each bimodal condition, analyzed separately for each preceding stimulus. Each row corresponds to a different preceding stimulus, labelled in the rightmost column. Color conventions same as Fig. 4.

As shown in Fig. 5, a similar pattern of race model violations was observed after controlling for sequence effects. In 15 out of 18 conditions (83.3% overall; AV: 6/6 [100%], AT: 4/6 [66.6%], VT: 5/6 [83.3%]), the fastest RTs significantly outpaced race model predictions in at least one time bin. In 18 out of 18 conditions (100%), the slowest RTs were significantly slower than race model predictions in at least 1 time bin. Averaged unimodal RT CDFs for each preceding stimulus are shown with corresponding bimodal RT CDFs in Supplemental Fig. S3.

To further examine the effects of preceding stimuli on the RTE, we computed the observed race model violation for each time bin by subtracting the observed RT CDF from the race model for each subject in each condition. To estimate the proportion of RTE variance explained by sequence effects, we fit separate within-subjects ANOVAs to each time bin (1400 bins) with a single factor for preceding stimulus. Then, for each stimulus condition (AV, AT, and VT), we added the sums of squares explained by the preceding stimulus across all time bins, and divided by the total (within-subjects) sums of squares across all time bins. This yielded a single value (R^2^) for the overall proportion of RTE variance explained by the preceding stimulus.

**Figure 6:**
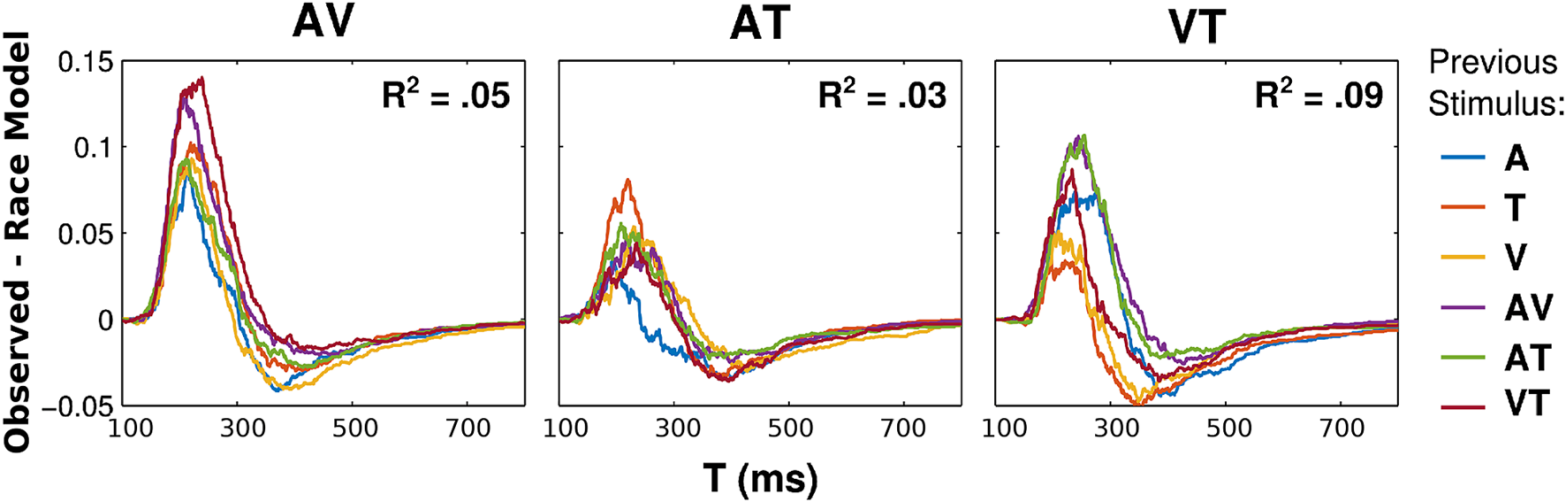
Race model violations (P[observed] − P[race model]) as a function of prior stimulus for each multisensory condition (AV, AT, and VT). Each curve is colored according to the previous stimulus (legend right). Effects of previous stimuli only explained a small proportion of variance (R^2^) across all time-bins.

As shown in Fig. 6, preceding stimuli only explained 3–9% (AV: 5.07%, AT: 3.01%, VT: 8.96%) of the variance in the observed RTE, and race model violations followed a similar qualitative pattern for each condition, regardless of the previous stimulus. Thus, taken together, our results suggest that preceding stimuli only have a modest effect on the size and shape of race model violations, and fail to account for general redundancy gain benefits.

### Model comparison

**Figure 7:**
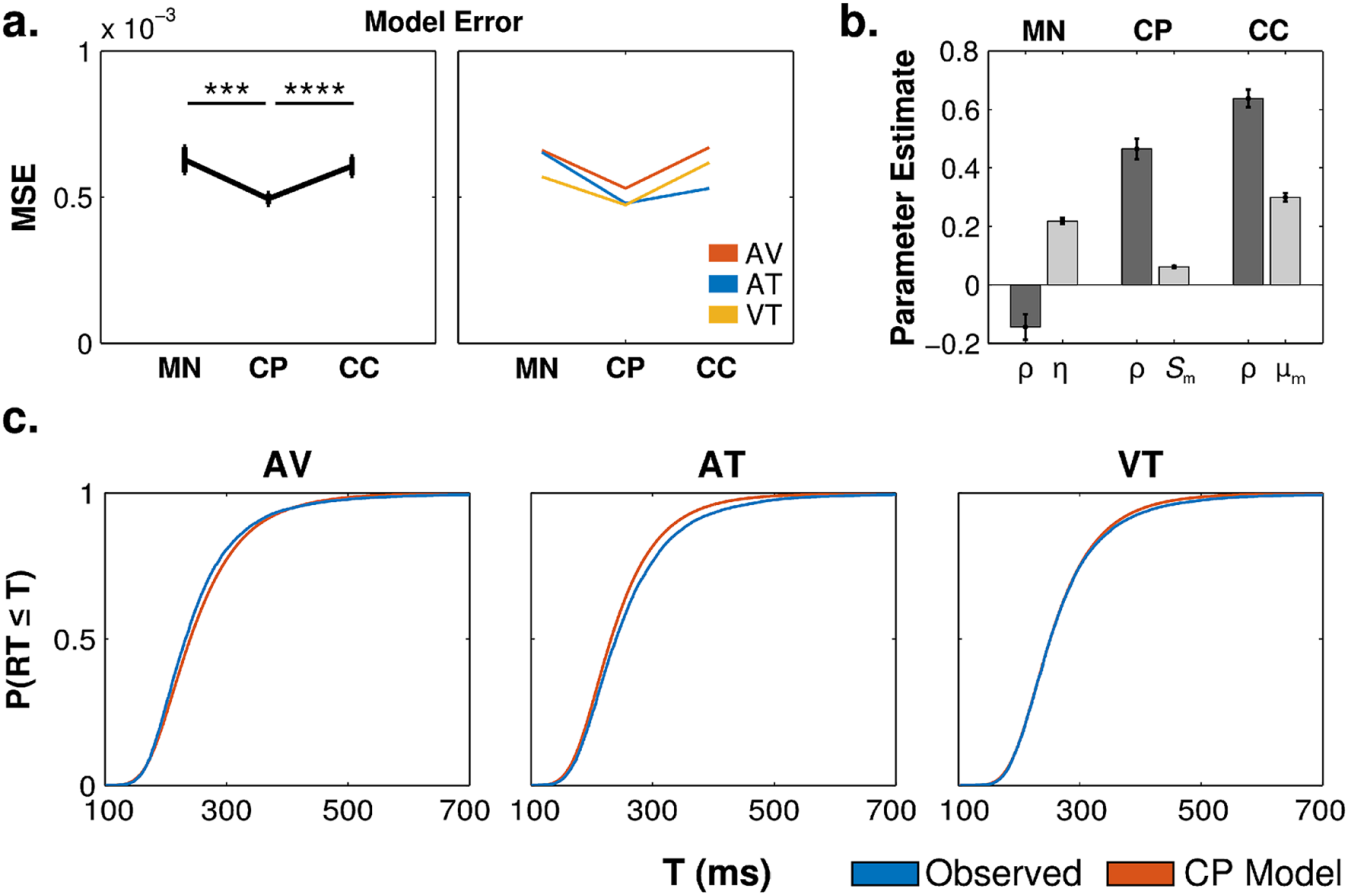
Multisensory model comparisons. (**a**) Mean squared error (MSE) for each model fit, averaged across subjects. The best-fitting CP model exhibited significantly less error than best-fitting MN and CC models. Error bars represent bootstrapped (10,000 samples) within-subjects SEM (i.e., the mean MSE for each subject was subtracted before sampling). (**b**) Best-fitting parameters for each model, averaged across subjects. Errors bars represent bootstrapped SEM (10,000 samples). (**c**) Empirical RT distributions (blue) and CP model predictions (orange), averaged across subjects.

To more precisely characterize multisensory interactions in the RTE, we compared the three models described above: the multisensory noise model (MN), the crossmodal pre-potentiation model (CP), and the crossmodal co-activation model (CC). Each model was fit to each subject's RT CDF for the AV, AT, and VT conditions, and we compared the average mean-squared error (MSE) across models using permutation tests (sign-flipping; 10,000 permutations). Averaging across the observed MSE for each bimodal condition, we found that the CP model produced significantly better fits than the MN (*p* < .005) and CC models (*p* = .001), with no significant difference between the MN and CC models (*p* = .78; Fig. 7a, left). The same pattern was evident in each condition, and there was no significant condition by model interaction (Fig. 7a, right).

The best fitting parameters for each model are shown in Fig. 7b. As expected, the MN model and CP model made opposing predictions about the intersensory correlation coefficient (dark gray bars). While the MN model was best fit with a negative correlation coefficient, both the CP and CC models were best fit with a positive correlation coefficient.

To illustrate the predictions of the best fitting CP model, we averaged the model predictions for each time bin across subjects (orange) and plotted it with the averaged bimodal RT CDFs for each condition (blue) in Fig. 7c. As can be appreciated from the figure, the model fits are qualitatively quite good throughout the RT distributions for each condition.

## Discussion

Here, we examined the distributions of multisensory RTs to assess multiple possible explanations for the redundant target effect (RTE). First, we replicated the previous observation that multisensory RT distributions both lead (on the left tail) and lag (on the right tail) race model predictions^9^. While the lagging of slower RTs has been less emphasized in studies of the RTE, capturing this unique feature of multisensory RT distributions is critical for any model of multisensory RTs.

Second, we found that, after controlling for lag-1 stimulus sequence effects, the same pattern was largely retained. For 15 out of 18 stimulus sequences (83.3%), the fastest RTs significantly outpaced race model predictions in at least one time bin, while the slowest RTs lagged model predictions for all 18 sequences (100%). Moreover, we observed significant race model violations (in both directions) for all conditions where modality switch costs would presumably affect both (e.g., AV following T) or neither (e.g., AV following AV) of the target stimulus modalities. Because neither irrelevant (e.g., T before AV) nor fully redundant (e.g., AV before AV) prior stimuli would be expected to selectively speed or slow processing in only one modality on subsequent bimodal trials, these race model violations are unlikely to be explained by negative intermodal correlations produced by modality switch costs. Thus, these results suggest that modality switch costs are unlikely to explain the RTE. Consistent with this interpretation, we found that the modality of the preceding stimulus only explained 3–9% of the variance in race model violations across the RT distribution.

These results stand in contrast to previous reports showing that the RTE may be abolished when switch costs are eliminated through task design. For example, Shaw et al.^10^ found that the audiovisual RTE was observed when stimuli were presented in random order, but was abolished when A, V, and AV stimuli were presented in separate blocks. One potential explanation is that blocked designs do not enforce attention across multiple senses during multisensory trials, potentially allowing participants to attend selectively to a preferred or more salient modality, thereby weakening multisensory interactions^35–39^. Indeed, consistent with previous reports^7,10^, Shaw et al. found that, when filtering for repeated AV trials within a mixed design, the RTE was weakened but still observed, calling into question an explanation based purely on switch costs. Taking all the evidence together, we consider it most likely that modality switch costs may influence, but not fully explain the apparent RTE, and that blocked designs may inadvertently suppress the multisensory interactions that subserve the effect.

This may also explain why the apparent influences of previous stimuli were particularly modest in the current study. Because the task required continuous monitoring of the auditory, visual, and tactile modalities for approximately equiprobable events, participants were encouraged to keep their attention “spread” across the senses, potentially facilitating multisensory interactions^35–39^.

Finally, we compared three multisensory extensions of the LATER model—the multisensory noise model (MN), the crossmodal pre-potentiation model (CP), and the crossmodal co-activation model (CC)—to identify which could best explain the unique shape of multisensory RT distributions. Consistent with our hypotheses based on the expected effects of phase-resetting on sensory evidence accumulation, we found that multisensory RT distributions were generally best fit by the CP model, in which multisensory interactions increase initial sensory evidence at stimulus onset, thereby pre-potentiating sensorimotor responses. This is consistent with the expected effect of crossmodal phase-resetting, which rapidly places sensory cortex in a high-excitability state before sensory input arrives^15–22^. Moreover, consistent with the fitted parameters of the CP model, crossmodal phase-resetting is expected to produce temporal dependencies across the senses through phase alignment. In total, these results support previous observations demonstrating correlations between crossmodal phase-resetting and the RTE^23,24^.

One potentially interesting implication of this result is that it provides a common mathematical framework in which both neurophysiological and behavioral indicators of multisensory integration can be considered. While currently the correspondences are primarily qualitative, future studies may be able to use similar models to more directly relate the effects of phase-resetting on neural evidence accumulation and subsequent behavior. In the LATER model, increasing the initial evidentiary state S_0_ is equivalent to increasing the prior log-odds of stimulus presence versus absence^14^. As explicated here (see Fig. 3), this results in different effects on evidence accumulation than increasing the accumulation rate. Thus, it is important to consider the extent to which multisensory interactions influence perceptual inference through the rapid modulation of perceptual priors, rather than convergence of sensory signals. Understanding how shifts in cortical excitability interact with thalamocortical input to impose these priors may prove important to understanding the computational basis of multisensory perception.

## Methods

### Participants

116 undergraduate students (age 18–25) from Northwestern University gave informed consent to participate in the experiment in exchange for $15 or partial course credit. All participants had normal hearing and normal or corrected-to-normal vision. For analysis, we included only those participants (N = 78) who responded within a predefined response window (100–1500 ms) on at least 80% of trials for each stimulus modality. This response window was chosen following Otto and Mamassian (2012). The experiment was approved by the Northwestern University Social and Behavioral Sciences IRB and performed while both authors were affiliated with Northwestern University. All study protocols adhered to relevant ethical guidelines/regulations.

### Materials

Testing was conducted in a lit, sound-attenuated room. Visual stimuli were presented on a 21-in color CRT monitor at a viewing distance of approximately 65 cm. Auditory stimuli were presented through Sennheiser HD 280 Pro (Old Lyme, CT) closed-back headphones. The headphones provided an estimated 32 dB attenuation of potential ambient sounds (manufacturer estimate). Tactile stimuli were delivered through two speaker cones enclosed in a plastic cube (~ 5 cm on each side). The speaker cones were exposed by two circular holes (~ 1 cm diameter) on opposite sides of the cube. Throughout the experiment, participants gripped the cube with their non-dominant hand, placing their thumb and middle finger over the circular holes. Participants responded with their dominant hand using a Cedrus Response Box model RB-834.

All stimuli were 100 ms in duration. The visual stimulus was a small (~ 0.75° visual angle) low luminance (19.4 cd/m^2^) disk presented in the center of a dark (2.5 cd/m^2^) screen. The auditory stimulus was a 3500 Hz pure tone (~ 40 dB SPL). The tactile stimulus was a 300 Hz triangle wave. Auditory white noise (~ 32 dB SPL) was played continuously throughout the task to ensure that participants could not hear the tactile stimulator. A small (< 0.1° visual angle) red fixation dot was presented in the center of the screen throughout the task. All stimuli were presented above detection threshold, as estimated by previous literature and confirmed in pilot testing (see^40^, Fig. 1, for review of detection thresholds for tones in continuous noise).

All stimuli were presented using Psychophysics Toolbox 3.0.14^41,42^. Stimulus timing was calibrated using a photometer and microphone to ensure that auditory, visual, and tactile stimuli were temporally aligned with less than 1 ms error.

### Experimental task design and procedure

Participants performed a speeded simple response time task in which they responded to auditory, visual, tactile, and bimodal (audiovisual, audiotactile, and visuotactile) stimuli. They were instructed to respond as fast as possible to stimuli in any modality while minimizing misses and false alarms. After seven practice trials, the task was completed in four blocks lasting approximately 15 min each (60 min in total). Each block consisted of 350 trials with a random inter-stimulus interval of 1500–2500 ms. In total, the task consisted of 1400 trials, including 212 trials for each stimulus type, and 128 catch trials.

Stimuli were presented in quasi-random order with the constraint that each possible two-stimulus sequence was presented at least 30 times. This was accomplished by randomly generating trial sequences until this constraint was satisfied. The selected sequence was then divided into four blocks which were presented in counterbalanced order across participants. See Supplemental Table 1 for the frequencies of each two-stimulus sequence.

### Computation

#### CDFs and race model

After rejecting responses outside of the 100–1500 ms response window (median: 2.4% rejected; range: 0.1–14.5%), we computed empirical CDFs with 1 ms bins for each stimulus type for each subject. For our initial analysis of the RTE, we used CDFs estimated from all trials, regardless of the preceding stimulus. For our analysis of the effects of previous stimuli, we computed separate CDFs for each two-stimulus sequence, using only trials with the same preceding stimulus for each CDF. Trials were partitioned based on the previously presented stimulus, regardless of whether participants responded to the previous stimulus within the response window.

Race models were estimated using the typical race model inequality under the assumption of statistically independent unimodal channels: $P\left( {RT \le T} \right) = P\left( {M_{1} } \right) + P\left( {M_{2} } \right) - P\left( {M_{1} } \right) \times P\left( {M_{2} } \right)$, where M_i_ represents the cumulative probability of a unimodal response in modality *i* for each time *T*. In our analysis of sequence effects, race model predictions were estimated using only unimodal trials with the same preceding stimulus as the bimodal CDF they were compared to. This ensured that potential effects of the preceding stimulus were incorporated into both the race model and empirical bimodal CDFs.

#### The LATER model and multisensory extensions

The LATER model predicts that unimodal RT distributions follow a reciprocal normal distribution,$\frac{\varTheta }{r}$, where Θ represents the distance between the initial evidentiary state, *S*_0_, and the decision threshold, *S*_T_, and the evidence accumulation rate, *r,* is normally distributed with mean μ and variance σ^2^. Thus, the LATER model can be expressed as: $RT \sim \frac{\varTheta }{{N\left( {\mu ,\sigma^{2} } \right)}}$, where Θ is set to 1 by convention.

The LATER model can be extended into a bimodal race model by replacing the denominator with the maximum function of two unisensory accumulation rates, *r*_1_ and *r*_2_: $\frac{\varTheta }{{max\left( {r_{1} ,r_{2} } \right)}}$^9^. Because both rate parameters are assumed to be normally distributed, the denominator, $max\left( {r_{1} ,r_{2} } \right)$, can be computed as the maximum function of a bivariate normal distribution with mean accumulation rates μ_1_ and μ_2_, variances σ^2^_1_ and σ^2^_2_, and correlation coefficient ρ. This gives the expected distribution of the maximum of two normally distributed variables (here, *r*_1_ and *r*_2_) with correlation ρ. This maximum function can be computed analytically using the equations derived in Nadarajah and Kotz^43^.

The multisensory noise (MN) model extends this bivariate LATER model by including an additional noise parameter, η, which adds additional variability to evidence accumulation rates beyond the variability estimated for each unisensory CDF. Thus, when each unimodal RT distribution is modelled as $RT_{i} \sim \frac{\varTheta }{{N\left( {\mu ,\sigma^{2} } \right)}}$, the combined bimodal distribution is estimated using $RT_{i} \sim \frac{\varTheta }{{N\left( {\mu ,\left( {\sigma + \eta } \right)^{2} } \right)}}$ for each modality, *i,* with η and the correlation coefficient, ρ, as free parameters. Thus, the η parameter represents additional variability in evidence accumulation rates that is unique to multisensory trials.

Importantly, however, the MN model does not account for the possibility that the distance-to-threshold parameter, Θ, or mean accumulation rate parameters, μ_1_ and μ_2_, could also be affected by multisensory interactions. In the MN model, Θ is fixed at 1 and each μ parameter is preserved from the unisensory LATER fits.

Here, we extended the bivariate LATER model to allow for changes in either Θ, μ, or σ on multisensory trials. As discussed above, changes in each of these parameters captures the primary predictions of a plausible model of multisensory interactions. Whereas increases in η represents increased variability on multisensory trials, decreases in Θ represent a decreased distance to the response threshold (consistent with crossmodal pre-potentiation; CP), and increases in μ represent an increased rate of evidence accumulation (consistent with crossmodal co-activation; CC).

In this generalized model, when each unimodal RT distribution is modeled as $RT_{i} \sim \frac{\varTheta }{{N\left( {\mu ,\sigma^{2} } \right)}}$, the combined bimodal distribution is estimated using $RT_{i} \sim \frac{{1 - S_{m} }}{{N\left( {\mu + r_{m} ,\left( {\sigma + \eta } \right)^{2} } \right)}}$ for each modality, *i.*

Just as the η parameter represents additional variability that is unique to multisensory trials, the variables *S*_m_ and μ_m_ represent changes in the distance-to-threshold and rate parameters, respectively, that are unique to multisensory trials. The variable *S*_m_ represents reductions in the distance-to-threshold parameter, Θ, relative to the unisensory models, in which Θ is fixed to 1 by convention. *S*_m_ can be thought of equivalently as either an increase in the initial evidentiary state, *S*_0_, or a decrease in the response threshold, *S*_*T*_. The variable μ_m_ represents increases in the mean rate of evidence accumulation relative to the unisensory models.

To compare the MN, CP, and CC models, we fit the generalized bivariate model to each subject's bimodal RT CDFs while allowing only one of the multisensory-specific parameters (η, *S*_m_, or μ_m_) and the correlation coefficient, ρ, to vary freely for each model, while the other parameters were fixed at zero. Therefore, the MN model had free parameters η and ρ, the CP model had free parameters *S*_m_ and ρ, and the CC model had free parameters μ_m_ and ρ.

Each model was fit to each subject's RT CDFs for the AV, AT, and VT conditions by minimizing the sum of squared errors using the fminsearch function in MATLAB R2014a (www.mathworks.com). Only time bins in which RTs were observed were included in model fitting.

## Supplementary Information

Supplementary Information.

## Data Availability

The datasets generated and analyzed during the current study are available in the Open Science Framework repository, https://osf.io/grckd/. This includes the individual rt and accuracy scores (1400/subject) from n = 78 subjects and the basic analysis scripts.
