# Supplementary material for: Multisensory stimuli shift perceptual priors to facilitate rapid behavior
Source: Sci Rep. 2021 Nov 29;11:23052. doi: 10.1038/s41598-021-02566-8 (PMC8629992; doi:10.1038/s41598-021-02566-8)
Supplement: Supplementary file 1 — Supplementary Information. [file 41598_2021_2566_MOESM1_ESM.pdf]

Multisensory Stimuli Shift Perceptual Priors to Facilitate Rapid Behavior

John Plass, David Brang*

Department of Psychology, University of Michigan, Ann Arbor, MI 48109, USA

*Corresponding Author: djbrang@umich.edu

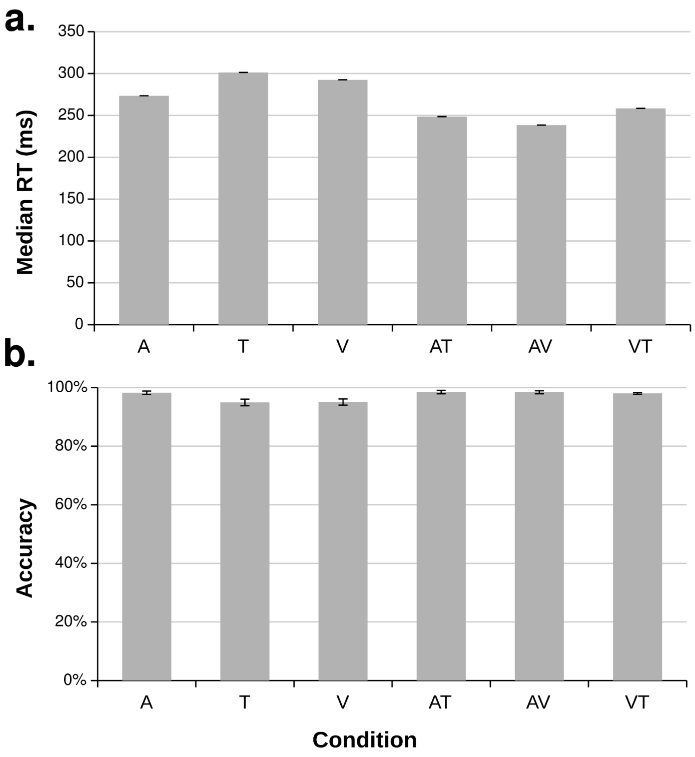

Figure S1. Median reaction time (A) and accuracy (B) averaged across participants (N=78) for each stimulus condition. Accuracy indicates the percentage of trials in which participants responded within the 100 - 1500 ms response window. Error bars indicate 95% confidence intervals.

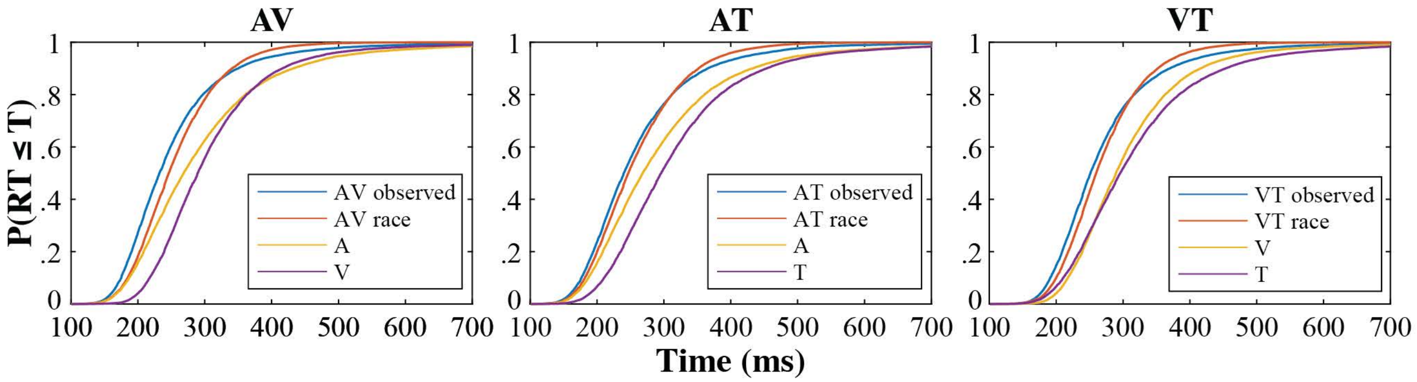

Figure S2. Comparisons of empirical RT distributions for unimodal (yellow and purple) and bimodal (blue) trials, along with race-model distributions (red), aggregated across all preceding stimuli.

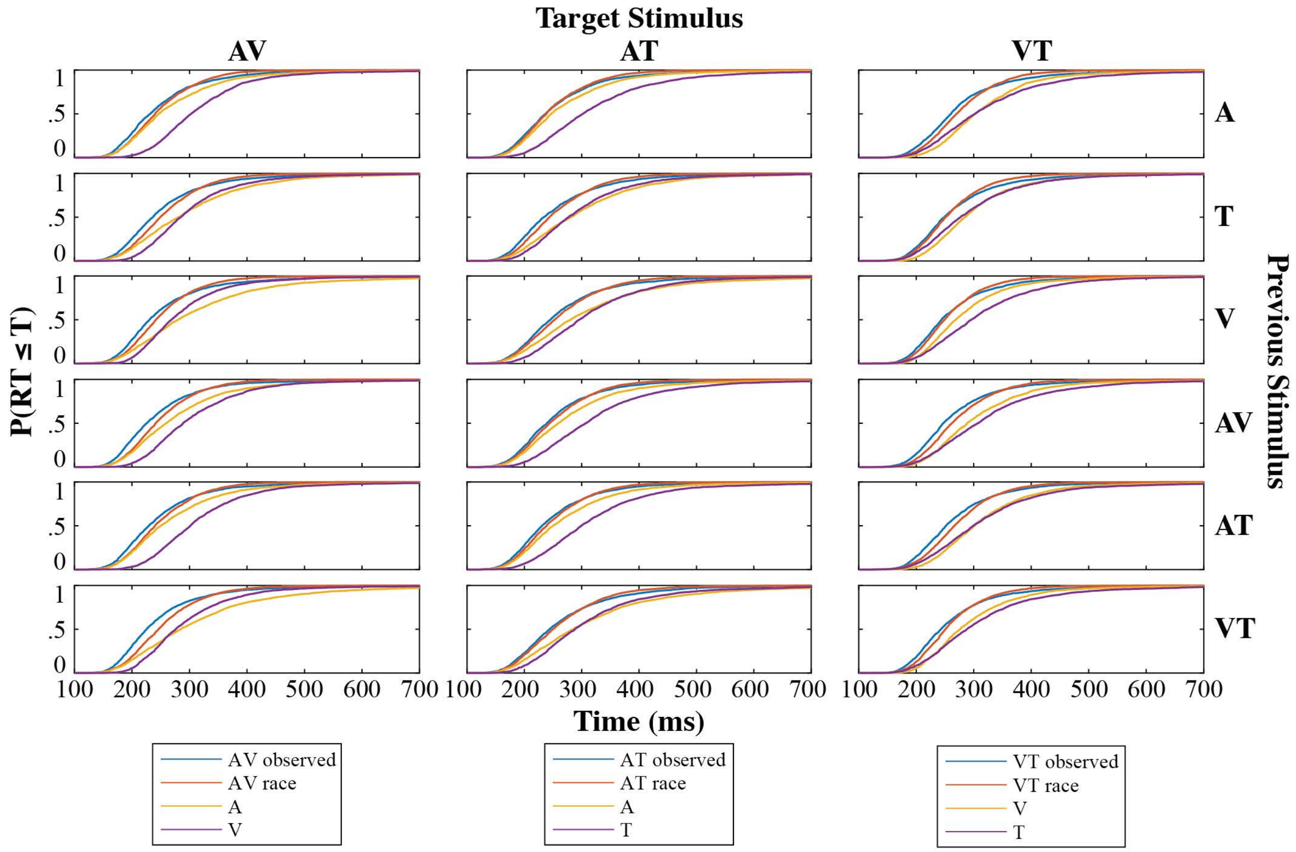

Figure S3. Comparisons of empirical RT distributions for unimodal (yellow and purple) and bimodal (blue) trials, along with race-model distributions (red), analyzed separately for each preceding stimulus. Each row corresponds to a different preceding stimulus, labeled in the rightmost column.

		Target Stimulus						
		**A**	**T**	**V**	**AT**	**AV**	**TV**	**Catch**
**Prior Stimulus**	**A**	32	32	31	32	31	31	23
	**T**	32	33	31	32	33	34	16
	**V**	34	32	33	33	32	33	14
	**AT**	31	31	34	32	32	32	19
	**AV**	31	34	32	33	32	32	18
	**TV**	31	31	32	35	31	32	20
	**Catch**	21	18	19	14	21	17	17

Supplemental Table 1. Frequencies of each two-trial sequence throughout the task. Columns indicate the modality of each target stimulus. Rows indicate the modality of each preceding stimulus.
